# Intestinal colonization with multidrug-resistant *Enterobacterales*: screening, epidemiology, clinical impact, and strategies to decolonize carriers

**DOI:** 10.1007/s10096-023-04548-2

**Published:** 2023-01-21

**Authors:** Edgar I. Campos-Madueno, Melika Moradi, Yasmine Eddoubaji, Fatemeh Shahi, Sina Moradi, Odette J. Bernasconi, Aline I. Moser, Andrea Endimiani

**Affiliations:** 1 Institute for Infectious Diseases, University of Bern, Bern, Switzerland; 2 Graduate School of Cellular and Biomedical Sciences, University of Bern, Bern, Switzerland; 3 Department of Microbiology, School of Medicine, Ahvaz Jundishapur University of Medical Sciences, Ahvaz, Iran; 4 Infectious and Tropical Diseases Research Center, Health Research Institute, Ahvaz Jundishapur University of Medical Sciences, Ahvaz, Iran; 5 Department of Computer Engineering and Information Technology, Islamic Azad University Tehran Central Branch, Tehran, Iran; 6 Institute of Medical Virology, University of Zurich, Zurich, Switzerland

**Keywords:** ESBL, SDD, FMT, Bacteriophages, Probiotics, Microcins

## Abstract

The clinical impact of infections due to extended-spectrum β-lactamase (ESBL)- and/or carbapenemase-producing *Enterobacterales* (*Ent*) has reached dramatic levels worldwide. Infections due to these multidrug-resistant (MDR) pathogens—especially *Escherichia coli* and *Klebsiella pneumoniae*—may originate from a prior asymptomatic intestinal colonization that could also favor transmission to other subjects. It is therefore desirable that gut carriers are rapidly identified to try preventing both the occurrence of serious endogenous infections and potential transmission. Together with the infection prevention and control countermeasures, any strategy capable of effectively eradicating the MDR-*Ent* from the intestinal tract would be desirable. In this narrative review, we present a summary of the different aspects linked to the intestinal colonization due to MDR-*Ent*. In particular, culture- and molecular-based screening techniques to identify carriers, data on prevalence and risk factors in different populations, clinical impact, length of colonization, and contribution to transmission in various settings will be overviewed. We will also discuss the standard strategies (selective digestive decontamination, fecal microbiota transplant) and those still in development (bacteriophages, probiotics, microcins, and CRISPR-Cas-based) that might be used to decolonize MDR-*Ent* carriers.

## Introduction

The global spread and continuous increase of multidrug-resistant (MDR) *Enterobacterales* (*Ent*) represent a serious concern for our health-care systems [1, 2]. These Gram-negative pathogens can be resistant to the commonly used third-generation cephalosporins (3GCs) and carbapenems, mainly due to the production of extended-spectrum β-lactamases (ESBL; e.g., CTX-M-types) and carbapenemases (e.g., KPC-, NDM-, and OXA-48-types), respectively. Moreover, since such ESBL and carbapenemase genes are carried by mobile-genetic elements (MGEs; e.g., plasmids) co-harboring other antimicrobial resistance genes (ARGs), these organisms are also frequently co-resistant to other classes of antibiotics, such as quinolones, aminoglycosides, and polymyxins [1–4]. Overall, this phenomenon drastically limits our treatment options [5]. As a result, infections caused by ESBL- (ESBL-*Ent*) or carbapenemase-producing *Ent* (CPE) are responsible for higher morbidity and mortality rates compared to those due to less resistant organisms [6, 7]. In 2018, this fact prompted the World Health Organization to classify both ESBL-*Ent* and CPE among the most critical priority pathogens for research and development of new therapeutic strategies and rapid diagnostics [8].

Since the *Ent* are common commensals of the intestinal microbiota [9], infections due to MDR-*Ent*—especially *Escherichia coli* (*Ec*) and *Klebsiella pneumoniae* (*Kp*)—may arise from a prior asymptomatic gut colonization [10, 11]. In addition, in both hospital and community settings, long-standing carriers can transmit such pathogens to other people in close contact with them [12]. Therefore, subjects colonized with MDR-*Ent* should be rapidly and accurately identified to implement infection prevention and control (IPC) measures [13, 14]. More importantly, eradication of MDR-*Ent* from the gut of carriers (“decolonization”) can represent an ideal clinical solution to prevent difficult-to-treat infections [15], but may also limit the epidemiological spread of these life-threatening bacteria in humans [16]. In this review, we present a summary of the different aspects linked to intestinal colonization due to MDR-*Ent*.

## Screening of carriers

In the hospital setting, screening for the intestinal carriage of MDR-*Ent* is an important procedure [13]. In fact, this approach is implemented with the aim to detect such carriage (e.g., patients with CPE) and prevent or contain the spread of MDR-*Ent* (e.g., occurrence of outbreaks) [14]. For instance, in many countries, such proactive procedures are routine when patients are transferred between hospitals or admitted from high prevalence areas [17–19]. However, also healthy individuals can be colonized with MDR-*Ent*. In fact, various surveys have shown that risk factors such as travel and previous antibiotic treatment greatly influence colonization [17, 20]. Therefore, not only subjects in healthcare-associated settings and in high-endemic regions, but also healthy people in the community can be screened to identify MDR-*Ent* carriers.

Screening of the intestinal flora for MDR-*Ent* (e.g., ESBL-*Ent* and/or CPE) is achieved by analyzing fecal samples (stool, perianal/perirectal, or rectal swabs) using culture- or molecular-based methods [13, 21]. A stool specimen is considered the gold standard, but perianal swabs are easier to obtain and less invasive than rectal swabs. Unfortunately, the performance of perianal swabs in detecting colonization with MDR-*Ent* has been investigated in only two comparative studies. Lautenbach et al. found 90% sensitivity of perianal swabs for detection of fluoroquinolone-resistant *Ec* [22]. In a larger study, Kubiak et al. found that perianal swabs had a concordance of ≥ 98% with stool in detecting ESBL-*Ent* [23]. Notably, in both studies, selective cultures were performed without broth pre-enrichments (see below).

### Culture-based methods

In culture-based methods, a fecal sample (~ 50-100 µg) is plated directly onto various commercially available selective chromogenic media. For instance, to detect carbapenem-resistant *Ent* (CRE), the CHROMID® CARBA or CARBA SMART (bioMérieux), and KPC or mSuperCARBA™ (CHROMagar™) are commonly used [13]. For the detection of 3GC- and colistin-resistant (COL-R) strains, many other media exist and their performances have been extensively discussed elsewhere (e.g., [24–27]). Importantly, the main advantages that chromogenic media offer are the simultaneous detection of resistance phenotypes and species-level differentiation (i.e., colonies of different colors). Alternatively, non-chromogenic media such as in MacConkey agar supplemented with antibiotic disks (e.g., ertapenem) can be used for the detection, for example, of CRE in stools [28].

Detection of MDR-*Ent* directly from fecal samples using chromogenic media has a typical turnaround time (TAT) of 18–20 h (overnight incubation), though the final antimicrobial susceptibility tests require at least a further day. This approach can detect organisms with variable limits of detection (LOD), which typically depend on several factors such as chromogenic media brand, species, resistance mechanisms (e.g., KPC- vs. OXA-48-type carbapenemase producers), and input (i.e., stool inoculum) [13]. Therefore, direct plating on chromogenic media may not be sufficient to detect low level carriage of MDR organisms (MDROs).

To overcome this issue, the performance (i.e., increase in detection) of direct plating can be optimized by an additional pre-enrichment step [29–35]. This step is usually achieved by overnight incubation in the presence of one or several antibiotics in liquid broth [29]. The benefit of pre-enrichment is well known, but not consistently implemented due to the extra hands-on time in the clinical laboratory. Importantly, the implementation of a pre-enrichment step offers an increase in the recovery of low carriage organisms below the LOD [29]. For instance, Rondinaud et al. showed that an overnight pre-enrichment (100 mg of stools in 10 mL of brain-heart infusion broth supplemented by 1.5 mg/L of cefotaxime) improved the detection of ESBL-*Ent* by 11.7% without decreasing specificity [36]. In another study involving 343 patients, the direct culture identified only 71.1% of the stools positive for ESBL-*Ent*, whereas using pre-enrichments in tryptic soy broth, MacConkey or MacConkey plus cefuroxime [32 mg/L] and vancomycin [64 mg/L] positive samples were 88.9%, 91.1%, and 91.1%, respectively [31].

### Molecular-based methods

Various nucleic acid-based methods exist for the detection of MDR-*Ent* directly from fecal samples. These include the real-time PCR- (quantitative PCR, qPCR)-based, which allow for ARGs identification from fecal genomic DNA. Alternative to qPCR, the loop-mediated isothermal amplification (LAMP) is not affected by PCR-inhibitors typically present in fecal DNA, and is a fast and cheaper substitute to screen for MDR-*Ent* carriage [21, 37–39].

The qPCR method has been integrated into several automated systems that offer minimal hands-on time and faster TAT compared to culture methods. For instance, a multiplex qPCR approach is used by the GeneXpert® platform (Cepheid), which can implement the Xpert® Carba-R assay for the detection of major carbapenemase genes in less than 1 h [40, 41]. Other similar rapid qPCR-based automated platforms designed to detect ESBL and/or carbapenemase genes are, for example, BD MAX™ Check-Points CPO® (Check-Points), LightMix® modular carbapenemase (Roche), CRE/ESBL ELITe MGB® (ELITechGroup), and Novodiag® CarbaR+ (Hologic) assays; all of which provide results in less than 3 h [42–46]. However, a major drawback of these automated systems is that they are generally unable to detect novel ARGs because they rely on predefined targets [13]. They also tend to be significantly more expensive than culture-based systems [47]. Nevertheless, in this context, we emphasize that the longer TAT of culture-based methods may actually imply higher overall healthcare costs (e.g., for unnecessarily preemptive isolation for non-colonized patients) [48].

Molecular-based methods offer equal or higher sensitivity as culture-methods, with LODs that are variable from method-to-method and usually gene-dependent [42, 44]. For instance, Nass et al. showed that an in-house qPCR designed to detect the *bla*_NDM-1_ carbapenemase gene in spiked stool had 100% sensitivity and specificity. Moreover, the LOD was 1–3 x 10^1^ colony forming unit (CFU)/mL, whereas that of CHROMagar™ KPC selective culture plates ranged between 10^1^ and 10^3^ CFU/mL [49]. When testing 128 clinal rectal swabs, the BD MAX™ Check-Points CPO® showed sensitivity and specificity of 92.8% and 97.8%, respectively. In particular, 5 samples identified as positive by culture (ChromID® CARBA SMART plates) and by Xpert® Carba-R were not detected by the Check-Points CPO® assay [45]. Compared to the broth pre-enrichment culture, GeneXpert® showed sensitivity and specificity of 100% and ≥ 99% for the detection of *bla*_KPC_ and *bla*_VIM_ carbapenemase genes in fecal samples. Moreover, the system was able to detect 100% of the samples spiked with *bla*_NDM_-positive *K. pneumoniae* strains at concentrations of 300 CFU/mL [41].

As in culture-based approaches, molecular-based methods can greatly benefit from an enrichment step. For example, Donà et al. demonstrated that detection of the COL resistance gene *mcr*-*1* by qPCR increased when selective broth enrichments were used compared with native stools [50]. Similarly, a study by Girlich et al. found that an enrichment step was necessary for the detection of an OXA-181-producing *Ec* (OXA-181-*Ec*) from a rectal swab, which was previously categorized as negative by the qPCR-based Cepheid Xpert® Carba-R system and when using direct plating [51]. Therefore, molecular-based systems may require an enrichment step to increase their sensitivity to detect low carriage of MDR-*Ent* in stool.

It should also be noted that these methods cannot distinguish between DNA from alive and dead bacteria, so the presence of viable MDR-*Ent* is overestimated (i.e., negative result in culture, but positive in molecular method) [52]. For instance, in a multisite study involving 383 patients, 4–5% of the fecal samples resulted positive for carbapenemase genes with the Cepheid Xpert® Carba-R system with a corresponding negative result with the standard reference culture (MacConkey broth containing 1 mg/L meropenem and subculture in a MacConkey agar plate with a 10 μg meropenem disk) [40].

## Epidemiology and risk factors

### Hospital setting

The gut flora is a rich, constant, and dynamic reservoir that has been shown to be the major source of MDR-*Ent* in hospitalized patients [10]. Moreover, it hinges on various predisposing factors such as underlying diseases, exposure to antibiotics, and use of medical devices (e.g., nasogastric tubes and urinary catheters). As a consequence, intensive care units (ICUs) represent the setting with the highest risk for colonization and cross-transmission of MDR-*Ent* between patients [53, 54].

Numerous investigations have analyzed the MDR-*Ent* gut carriage in hospitalized patients. Depending on (i) the geographic region, (ii) its epidemiological situation (e.g., low-prevalence vs. endemicity), and (iii) the type of admission ward, very different prevalence data have been reported. In contrast, similar risk factors linked to the acquisition of the MDR-*Ent* are described. For instance, in the general population of hospitalized patients, the rates of MDR-*Ent* colonization (ESBL-*Ent* and CRE) ranged between 12 and 65% in different countries. However, history of antibiotic use, duration of hospital stay, nursing home residency, receiving parenteral nutrition, and previous hospital admission(s) were constantly recognized as independent factors associated with the carriage [55–59].

For ICU-patients, ESBL-*Ent* carriage rates of 62.3%, 8%, and 5.3% were recorded during 2014–2015 in Thailand, Switzerland, and France, respectively [60–62]. In Spain (period 2012–2013), 16% of the ICU-patients carried CPE [mostly OXA-48-producing *Kp* (OXA-48-*Kp*)], and the main risk factors associated with this condition were chronic renal disease, previous digestive/biliary endoscopy, hospitalization(s), intra-abdominal surgery, antibiotic use, and higher mortality prediction scores (e.g., median APACHE II score of 15) [63].

Gut colonization with MDR-*Ent* may also frequently involve pediatric patients. In Tanzania (2017–2018), Tunisia (2015), Gabon (2010–2011), and Cambodia (2012), 56%, 28.6%, 45%, and 55% of the hospitalized children were carriers of ESBL-*Ent*, respectively [64–67]. In Serbia (2017–2018), gut carriage with ESBL-producing *Kp* (ESBL-*Kp*) or ESBL-producing *Ec* (ESBL-*Ec*) was recorded in 59% of the hospitalized pre-term neonates; previous hospitalization, delivery by cesarean section, and mechanical ventilation were associated with colonization [68]. In a study from Morocco (2013–2015), up to 59.4% and 12.5% of the neonatal ICU (NICU)-patients were colonized with ESBL-*Ent* and CPE, respectively [69]. CPE carriage was also observed in 8.6% of inpatients in a pediatric hospital in China (2019), with those colonized having a history of invasive procedures and antibiotic exposures [70].

#### Long-term care facilities and nursing homes

People residing in chronic care facilities are at increased risk of gut colonization with MDROs, but the estimated prevalence varies between countries [71, 72]. For instance, in Switzerland (2010–2020), 10.5% of the long-term care facilities (LTCF) residents were colonized with ESBL-*Ec*, of which 58% belonged to the pandemic sequence type (ST) 131 lineage [73]. Consistent results were obtained in a more recent national Swiss study (2019), with an ESBL-*Ent* carriage of 11.6% and again a high prevalence of ST131 *Ec* strains [74]. In an Italian study (2008), 64% of the LTCF residents were colonized with ESBL-*Ent*, while 6.3% had CPE. Risk factors for colonization included age ≥ 86 years, antibiotic treatment in the previous 3 months, indwelling devices, chronic obstructive pulmonary disease, and physical disability [75].

In French nursing homes (2017–2018), 19.8% of the patients were colonized with ESBL-*Ent*, whereas CPE were not detected; use of a shared bathroom, previous antibiotic use and recent history of hospitalization were risk factors for colonization [76]. Similarly, in Belgium (2015) and California (2016–2017), 11.3% and 16% of the nursing home residents were gut carriers of ESBL-*Ent*, respectively [77, 78]. In Japan (2015–2017), this prevalence was instead as high as 36% [79].

### Community setting

The prevalence of MDR-*Ent* among healthy people in the community has reached alarming levels and now represents one of the most important threats to public health [80]. In the 1990s, MDROs were mainly associated with nosocomial infections. Since then, however, there has been an emergence and dissemination outside the hospital context, leading to an increase in infections due to these pathogens [81]. In particular, community-onset infections due to ESBL-*Ent* were increasingly being reported in the early to mid-2000s, while reports about community-associated CRE infections started to emerge around 2010 [82, 83]. More recently, there have also been an increasing number of reports for community-associated COL-R isolates that simultaneously possessed resistance mechanisms against other antimicrobials such as carbapenems, 3GCs, and aminoglycosides [84–86].

While intestinal colonization with MDR-*Ent* has been reported worldwide, the prevalence among the healthy population varies greatly between different regions. In a meta-analysis by Bezabih et al. regarding ESBL-*Ec*, the average prevalence for intestinal colonization ranged from 6% in Europe to around 20% in the Eastern Mediterranean and Africa, while it was up to 24.5% and 27% for the Western Pacific and South-East Asia, respectively [87]. However, for some countries, the reported numbers were much higher, with low-income countries usually showing a higher prevalence. For instance, studies from Tanzania (2018), Laos (2018), and Thailand (2010) reported rates of ESBL-*Ent* carriage in the healthy population as high as 91.5%, 70%, and 69.3%, respectively [88–90]. Though in high-income Asian countries such as Japan, the prevalence for ESBL-*Ec* in 2011–2012 (8.5%) was comparable to those reported for European countries (e.g., 7.1% in Switzerland in 2013–2016) [57, 91]. Furthermore, Bezabih et al. observed a yearly increase of 1.5% in the prevalence of ESBL-*Ec* with an estimated global prevalence of nearly 30% in 2020 [87]. This development is also reflected in a study conducted by French researchers who observed a 10-fold increase in the prevalence of ESBL-*Ec* in healthy subjects living in Paris from 2006 (0.6%) to 2011 (6.1%) [92]. A few studies have also indicated a high prevalence of healthy children in the community who are colonized with ESBL-*Ent*, such as ~ 5% in France (2010–2011), the Netherlands (2010–2012), and the USA (2013–2015), ~ 13% in Libya (2007), ~ 22% in Iran (2017), and 43% in Pakistan (2016) [93–98].

During 2016–2019, the prevalence of COL-R-*Ent* carriage among healthy people ranged from 2 to 3% in studies conducted in Taiwan, Spain (considering health-care workers, HCWs), and South Africa (considering children), while it was 15% in two studies that analyzed healthy Chinese and Laotian people in 2016 and 2018–2019, respectively [89, 99–102]. In contrast, a study from Bolivia (2016) and one from Vietnam (2017–2018) reported a wide dissemination of COL-R bacteria in the healthy community with rates of 38.3% and 70.4%, respectively [103, 104]. From the 70.4% of COL-R-*Ec* in Vietnam, the majority (92.8%) were also MDR. In both studies, the authors discussed the high amounts of COL used for animal breeding as a possible explanation for the high dissemination among the healthy community [103, 104]. In line with this, a study from China observed a decrease in *Ec* carrying the COL resistance gene *mcr* in the gut of healthy adults from 11.5% in 2018 to 2.4% in 2019 following the ban of COL as a growth promoter in animal breeding in 2017 [105].

CRE are also reported to colonize the intestinal tract of healthy individuals. In Cambodia (2011), Switzerland (2014), and India (2015–2017), CRE were detected in 1%, 0.1%, and 6.4% of the healthy population, respectively [106–108]. In Lebanon (2018), researchers found 6% of healthy bakery workers to carry CR-*Ec* [109]. Likewise, in the Eastern Mediterranean, a study conducted in Kuwait (2016–2018) found 7.7% of people working in the food industry to carry CRE, while 30.5% of the *Ent* isolates were also MDR [110].

With regard to the risk factors for people in the community, in some studies, regular contact with children and animals, and consumption of contaminated food (e.g., meat products and aquatic food) have been identified as risk factors for acquiring MDR-*Ent* [99, 100, 105, 106, 111–114]. More importantly, international travel (see below), previous hospitalization, and general health status (e.g., underlying disease, extreme age group, body mass index ≥ 25 kg/m^2^) are significantly associated with the carriage of MDR bacteria (e.g., [67, 111, 115]). For instance, HIV-positive individuals are at increased risk for acquiring MDR-*Ent* [116]. Of note, in such group of individuals, those receiving suppressive antiretroviral therapy (ART) appear to acquire MDR-*Ent* as likely as the general population. In the Swiss HIV cohort (2015–2016), the prevalence of 3GC-R-*Ent* carriers was found to be 6.7% [117], which is consistent with the rate found in healthy people (7.1%) during the same period [91]. In contrast, in low-income countries, subjects not receiving ART showed higher MDR-*Ent* colonization rates (e.g., 23–33% in Tanzania) than the general population [118, 119]. Interestingly, in a recent analysis, carriage of ESBL-*Ent* was more frequent in men who have sex with men undergoing preexposure prophylaxis or living with HIV-positives and with high number of sexual partners [120].

Concerning the transmission from animals, exposure to livestock, their manure, and slaughter products were found to be significantly associated to ESBL-*Ent* carriage [106, 121]. A Dutch study from 2020 that analyzed 3GC-R-*Ec* by whole-genome sequencing (WGS) confirmed the transmission between broilers and people working and living on farms in six cases [122]. Similarly, a study from Thailand (2018) applied a WGS approach to analyze ESBL- and CR-*Kp* and found the same clones in pigs and farmers, suggesting a direct transmission between the two groups [123]. In contrast, transmission from pets to humans seems to be less common. A recent meta-analysis found no significantly higher risk for carriage of 3GC-R-*Ent* in pet owners compared to non-pet owners [124]. In line with this, co-carriage of ESBL-*Ent* between owner and pet was rare in a Dutch study performed in 2020, with only 5 cases detected out of 550 analyzed pet-owner pairs [125]. Likewise, in a Swiss study (2016) in which 72 owners and their pets were screened for ESBL-*Ec*, only one case of direct transmission was detected, whereas in a more recent Swiss study (2021), no co-carriage of MDR-*Ent* was detected in 50 pet-owner pairs [126, 127].

#### Health-care workers

Health-care workers (HCWs) represent a special population in the community that may have a significantly different colonization prevalence with MDR-*Ent.* For instance, the prevalence of HCWs colonized with 3GC-R-*Ec* was 4%, 12%, 24%, 47%, 65%, and > 75% in Germany (2013–2014), Italy (2016), Egypt (2013), Rwanda (2014), Vietnam (2019), and Madagascar (2014–2015), respectively [55, 128–132]. Nevertheless, such prevalence seems consistent to that recorded in the general population (see previous section). Moreover, the above studies did not establish a clear association (transmission event) between MDR-*Ent* isolated from HCWs and MDR-*Ent* isolated from the patients at the same institutions. In fact, only the few surveys that have implemented adequate molecular techniques (e.g., pulse field gel electrophoresis, PFGE; multi-locus sequence typing, MLST; and WGS), are able to address this issue [133].

Among them, the MOSAR study (2008–2011) indicated that 3.5% of the HCWs of five rehabilitation units located in Israel, Italy, France, and Spain were gut colonized with ESBL-*Ent* (mostly ESBL-*Ec*); feeding patients was associated with carriage. However, only 1/3rd of the ESBL-*Ec* from the medical staff were actually molecularly linked (i.e., identical or highly-related clones) to those from their patients [134]. In another Spanish study (2018) involving 6 hospitals, only 3.1% HCWs resulted colonized with ESBL-*Ent*. No statistically significant risk factors for colonization were identified; more importantly, the rate of colonization was not higher than that reported for healthy people in the corresponding community [101]. In a Swiss study conducted in 4 veterinary institutions (2018), only 2 out of 108 (1.9%) HCWs resulted colonized with hyperepidemic clones of CP-*Ec* (i.e., ST410 producing OXA-181 and ST167 producing NDM-5); however, these CP isolates were molecularly identical to those frequently found among dogs and cats hospitalized at the same institutions [135].

Overall, the above studies seem to indicate that HCWs have a low risk of being colonized with the same MDR-*Ent* affecting their patients. Nevertheless, data on this context are still scarce, as emphasized by the systematic analysis of Peters et al. [136]. Therefore, further high-quality research is needed to assess the risk of occupational colonization with MDR-*Ent*.

### International travelers

One of the main risk factors for the acquisition of MDROs in the community of low prevalence areas is travelling to endemic countries [137–141]. In particular, travels to Asia and Africa have been associated with a high risk for MDR-*Ent* acquisition, especially ESBL-*Ec* [112, 138, 142–145]. The use of antibiotics during the trip can further contribute to an enhanced risk for colonization [112, 137].

In the COMBAT study (2012–2013), 34.3% of the overall Dutch tourists acquired ESBL-*Ent* when traveling abroad, but for those visiting southern Asia was 75.1%. Regarding the sub-group of travelers visiting the African continent, the acquisition rates were 18.9%, 27.8%, and 42% for Western Africa, Middle and Eastern Africa, and Northern Africa, respectively [146]. In another Swiss study conducted at the same time, 69.4% of all travelers from Switzerland to the Indian subcontinent returned colonized with ESBL-*Ec*, but those specifically returning from India had a colonization rate of 86.8% [144]. In a more recent study (2018–2019), we observed that 54% of Swiss travelers to Tanzania acquired MDR-*Ent*, of which 54% were ESBL-*Ec* and 16.2% were COL-R-*Ec.* Such MDR-*Ent* had a corresponding (identical) strain among resident people, food, animal and/or environmental sources [139]. COL-R-*Ec* strains possessing the *mcr*-*1* gene or chromosomal mechanisms were also isolated in 10.5% of the stool of Swiss travelers returning from India in 2015 [141]. Finally, we note that the isolation of CPE in returning travelers is still very rare, but their importation to low prevalence countries is a concern [144, 147].

## Length of colonization and spontaneous decolonization

The duration of intestinal colonization due to MDR-*Ent* has been analyzed in several studies involving adult patients hospitalized in acute institutions or admitted to various types of LTCFs. In contrast, data regarding healthy people in the community are scarce, with most of the surveys performed on international travelers.

### Hospitalized patients

In general, hospitalized people tend to remain colonized with MDR-*Ent* for the duration of their nosocomial stay, and approximately 50% of them show spontaneous decolonization without intervention within 6 months of discharge. However, this phenomenon occurs over a broad timeframe and depends on many factors (see examples below) [148, 149]. In addition, 15–45% of patients may retest positive after multiple negative screenings [150, 151]. This last phenomenon has important clinical implications (e.g., isolation of patients) and can be possibly explained in two ways: (i) the MDR-*Ent* was not eliminated from the intestinal tract, but only suppressed at a concentration below the LOD for the screening method used [13, 29, 31, 36, 50]; (ii) patients were actually decolonized, but re-acquired the MDR-*Ent* because they were exposed to the same environment, interventions, and/or treatments. In this context, the number of consecutive negative tests needed to define the eradication of intestinal colonization is essential, though standard criteria have not yet been defined in this regard [149]. Basically, eradication rates may be higher when a single sample defines the end of carriage than when multiple negative samples are required. Another issue is that most studies fail to demonstrate the persistence of the identical MDR-*Ent* using WGS techniques [149].

Several studies have well-summarized the above concepts [148, 149]. For instance, the meta-analysis by Bar-Yoseph et al. found that in the healthcare setting, 77% of colonized patients were still carriers of MDR-*Ent* at 1 month, 75% at 3 months, 55% at 6 months, and 35% at 12 months [149]. In a 14-year French study (1997–2010), 40% of readmitted patients with prior ESBL-*Ent* carriage were still colonized [152]. During an outbreak of KPC-2-producing *Kp* (KPC-2-*Kp*) in Germany (2010–2013), Lübbert et al. analyzed the gut carrier prevalence of adult patients by implementing both culture screening and a *bla*_KPC_-targeted PCR approach. Resolution of carriage was defined as at least 3 consecutive negative PCR tests at least 48 h apart. As a result, 69% of colonized patients tested positive after 1 month, 59% after 3 months, 35% after 6 months, 26% after 1 year, and 17% after 2 years. Of note, two patients retested positive for KPC-2-*Kp* after they had previously shown 3 consecutive negative tests, while one patient was colonized for 1191 days. The majority of patients who experienced spontaneous decolonization were those discharged from the hospital, whereas those who were long-term colonized usually had prolonged or repeated hospitalizations [153]. In a similar study, the multivariable logistic analysis performed by Kim et al. indicated that during 2015–2016, readmission [odds ratio (OR) = 9.96], carbapenem use (OR = 9.15), positive culture for a clinical sample (OR = 6.26), and duration of hospitalization (OR = 1.03) were predictive for persistent carriage of KPC-*Kp* after 6 months [154]. In another analysis (2013–2018), the same authors also indicated that CP-*Kp* may have a higher probability of prolonged carriage than other species of CPE. Furthermore, OXA-48-like-*Ent* showed a significantly increased risk of prolonged carriage than those producing NDMs; there was no significant difference between OXA-48-like and KPC producers [155].

### Pediatric patients

Löhr et al. investigated the duration of fecal carriage with ST17 and ST485 CTX-M-15-producing *Kp* (CTX-M-15-*Kp*) in infants colonized during a NICU outbreak (2008–2009) in Norway. The median carriage duration in infants after discharge was 12.5 months (the longest was 23.5 months). Risk factors for prolonged carriage were delivery by caesarean section and treatment with antibiotics during hospitalization [156]. Nordberg et al. performed a prospective cohort study (2008–2015) on 13 neonates colonized with an ST101 CTX-M-15-*Kp* responsible for an outbreak in two Sweden NICUs. As a result, the MDR pathogen was still found in two children at 23 and 26 months [157].

### Long-term residents

Unlike hospitalized patients, intestinal colonization with MDR-*Ent* in subjects admitted to long-term institutions can last for months. During 2013–2019, in a Dutch nursing home with an unusually high prevalence of rectal ESBL-*Ec* carriage, the colonization dynamics of ST131 ESBL-*Ec* vs. non-ST131 strains were evaluated. Spontaneous decolonization was observed in 33% of the ST131 carriers vs. 62% of those with other STs (*P* = 0.03). Survival analysis to calculate the median time to clearance showed that the half-life of carriage for the ST131 was 13 months, whereas only 2–3 months for other lineages (*P* < 0.001) [158].

### General population in the community

In a Dutch analysis (2014–2015), following a cross-sectional study (sample time T0), a subset of ESBL-*Ec*/ESBL-*Kp* gut carriers (*n* = 76) and non-carriers (*n* = 249) volunteered to provide 5 fecal swabs with an interval of 1 month (sample times T1 to T5). The median time between T0 and T1 was 125 days (range, 71–234 days). Of the initially positive participants (colonized), 25 (32.9%) remained positive in all subsequent samples (> 8 months), while 31 (12.4%) of initially negative individuals acquired ESBL-*Ec*/ESBL-*Kp* strains. Colonized subjects often carried the same *bla*_ESBL_ gene and plasmid, but sometimes in different host strains, indicative for horizontal gene transfer of MGEs (plasmids). Prolonged carriage was significantly associated with travel to countries with a high-prevalence of ESBL producers and being colonized with *Ec* strains of (i) phylogenetic groups B2/D, (ii) ST131, and (iii) producing CTX-M-9 group ESBLs [159].

### International travelers

Several cohort surveys conducted on healthy travelers assessed the MDR-*Ent* intestinal carriage among positive subjects after returning home. The VOYAG-R and the COMBAT studies (both in 2012–2013) reported that 10–25%, 5–14%, and 2–11% of the travelers returning colonized with MDR-*Ent* were still colonized with MDR-*Ent* at the 3-, 6-, and 12-month follow-ups, respectively [146, 160]. Other similar studies reported that after 6 months, 20–28% of the travelers who tested positive upon return were still colonized with MDR-*Ent* [140, 161–163]. Overall, these figures indicated that only 6 months after returning travelers have similar colonization rates to the non-traveling general population in high-income countries (e.g., 3–6% in Europe and North America) [20, 91]. Therefore, traveling abroad can be considered an additional risk factor for infection and/or transmission of MDR-*Ent* in the first 6 months upon return.

Some studies have also tried to assess the factors associated with sustained carriage in post-trip subjects. The VOYAG-R study revealed that carriage duration increased with travel destination, with Asia representing a higher risk compared to Africa and Latin America. This phenomenon might be linked to the higher concentration of MDR-*Ent* in the intestinal tract of people returning from Asia than those traveling back from other continents [160]. The COMBAT study reported that carriage of CTX-Ms-*Kp* and traveling to the Middle East were associated with a shorter carriage duration [146]. In another analysis, Armand-Lefèvre et al. suggested that long-term gut carriage in post-travelers is primarily due to the acquisition of specific epidemic clones of *Ec* (e.g., ST10, ST14, ST38, ST69, ST131, and ST648) that provide good adaptation to the human intestinal microbiota [164]. In our analyses, we noted that travelers may carry a median of 2 MDR-*Ent* clones (range 1 to 5) and that prolonged colonization in the follow-up period is due to clonal persistence or presence of the same plasmid in a new bacterial host [163]. Moreover, no specific microbiota patterns before travel were significantly associated with a higher risk of 3GC-R-*Ent* colonization [140]. In contrast, Peng et al. suggested that having low *Actinobacteria* richness and low abundance of short-chain fatty acid-producing bacteria in the gut microbiota may increase the risk of acquiring ESBL-*Ent* [165].

## Impact of colonization

### Clinical impact

As discussed above, MDR-*Ent* gut colonization is increasing in many settings. Thus, clinicians have to consider the risk of endogenous infections due to these difficult to treat pathogens. In this context, we emphasize that infections due to MDR-*Ent* are associated with higher health-care costs, morbidity and mortality [6, 7].

Numerous studies have indicated an association between previous gut colonization and infection due to MDR-*Ent*. However, this association seems to depend on the type of patients. For instance, Reddy et al. noted that 8.5% of the patients colonized with ESBL-*Ent* and admitted to high-risk wards during 2000–2005 developed a subsequent bloodstream infection (BSI) due to the same organism [166]. In a prospective analysis at three ICUs, Christiaens et al. reported that 69% of patients that were gut colonized with ESBL-*Ent* also had an infection or colonization with these organisms in another body site; in contrast, this was observed only in 12% of non-colonized subjects [167]. Another prospective study (2011–2012) with 497 hematological patients identified previous colonization as the most important risk factor (OR = 52) for BSI due to ESBL-*Ent* [168]. In a Swedish analysis considering a general hospitalized population (2004–2014), 6% of the gut carriers of ESBL-*Ent* developed an infection, but only 0.7% of them had a BSI [169]. This seems to support the hypothesis that gut colonization with MDR-*Ent* is a risk factor for BSI only in compromised patients. In a German study (2014–2015), 2386 ESBL-*Ec* and 585 ESBL-*Kp* rectal carriers admitted to a tertiary care centre were analyzed prospectively. Authors noted that the medical conditions of patients colonized with ESBL-*Kp* were more severe than those of patients colonized with ESBL-*Ec*. Moreover, a hospital-acquired infection (HAI) was observed in 7.8% and 13.8% following gut colonization with ESBL-*Ec* and ESBL-*Kp*, respectively. The most frequent types of infections were urinary tract infections (UTIs), surgical site infections, and BSIs. Patients colonized with ESBL-*Kp* had a significantly higher risk of developing HAIs with these pathogens than patients colonized with ESBL-*Ec* [relative risk (RR) = 1.62; *P* = 0.020] [10].

In the community, travel-related gut colonization with MDR-*Ent* seems to represent a non-negligible risk factor for infection, especially for UTIs [11, 170]. Several studies indicated that having traveled abroad (especially to Asian countries) within the year prior to symptoms is a 4- to 14-fold risk factor for UTI caused by an ESBL-*Ec* [171–174]; this is also true for children (OR = 8.93) during the first 6 months after the trip [175]. Moreover, Soraas et al. reported a 21-fold risk of UTI in adults when a shorter period of 6 weeks after travel was considered [176]. However, for all of these studies, analysis of fecal samples was not performed. Therefore, a definite link between previous gut colonization and subsequent UTI cannot be established.

### Contribution to the transmission of MDR-*Ent*

In a survey performed at our institution (2008–2010), index patients with carriage of ESBL-*Ec* or ESBL-*Kp* (mostly CTX-M-15 producers) were prospectively analyzed together with their hospital and household contacts after discharge. Hospital transmission rates were 4.5% and 8.3% for ESBL-*Ec* and ESBL-*Kp*, respectively. Incidence of ESBL-*Kp* hospital transmission was significantly higher than that of ESBL-*Ec* (*P* < 0.0001) despite the implementation of IPC measures. In the households, transmission rates were 23% for ESBL-*Ec* and 25% for ESBL-*Kp*, indicating that this setting exceed the nosocomial for the transmission of ESBL producers [12]. In another study (2008–2009), transmission from infants colonized with CTX-M-15-*Kp* during an NICU outbreak to parents/relatives was also observed in 32% of the households [156]. It should also be noted that there are numerous accounts in the literature of colonized patients hospitalized in other institutions (including abroad) who imported MDR-*Ent* to low-prevalence countries [177–181]*.* These patients can transmit their MDR-*Ent* and consequently generate outbreaks. Paradigmatic examples of this phenomenon are those associated to the importation of KPC-*Kp* (e.g., [182, 183]).

With regard to the LTCF setting, data are scarce. In a Dutch analysis performed in 2013–2014, transmission rates of ST131 ESBL-*Ec* were comparable, or even lower, than those of ESBL-*Ec* belonging to other lineages [184]. In another survey at a French LTCF (2009), patients and hospital staff carried a wearable sensor to monitor their interactions over a 4-month period. As a result, it was shown that ESBL-*Kp* can spread between individuals during close-proximity interactions, whereas this was not the case for ESBL-*Ec*, suggesting that only ESBL-*Kp* should be controlled by contact reduction interventions [185].

In the community setting, Valverde et al. reported that in 2004–2005, Spanish people with a community-acquired infection (mostly UTI) and their household members represented a reservoir for ESBL-*Ec*. In particular, 70% and 17% of the patients and relatives were colonized at gut level with ESBL-*Ec*, respectively. Moreover, 66% of the strains isolated from both groups were indistinguishable by implementing the PFGE analysis [186]. Transmission of MDR-*Ent* may also occur between returning travelers, who are gut colonized, and their household contacts, though data on this aspect are scarce. In the COMBAT study, a transmission rate of 4.7% was observed between positive travelers upon return and members of the same household who had not traveled [146].

## Strategies to decolonize carriers

Since colonized patients are at high-risk of developing severe infections, there have been numerous attempts to eradicate MDR-*Ent* gut carriage. In a systematic review performed in 2019 by the ESCMID–EUCIC (European Society of Clinical Microbiology and Infectious Diseases–European Committee on Infection Control), authors analyzed the available literature (i.e., 27 studies) regarding the strategies to decolonize gut carriers of MDR Gram-negatives. As a result, it was not recommended the routine use of interventions aimed at achieving decolonization from 3GC-R-*Ent* and CRE. However, these guidelines were mainly based on studies implementing the selective digestive decontamination (SDD) with oral antibiotics. Moreover, for fecal microbiota transplantation (FMT), authors did not provide recommendations due to the scarcity of data [187].

In this section, we will provide an overview of the main strategies used to attempt to decolonize MDR-*Ent* gut carriers. We will also analyze the novel and alternative approaches that may be developed in the near future.

### Selective digestive decontamination with antibiotics

Selective decontamination of the digestive tract (SDD) and selective oropharyngeal decontamination (SOD) are prophylactic antibiotic interventions for patients colonized with *Staphylococcus aureus* or aerobic Gram-negative bacteria. Most studies investigate the effectiveness of SDD and SOD in immunocompromised or critically ill patients [187, 188].

SDD includes topical antibiotics applied to the mouth and stomach, whereas in SOD, antibiotics are applied only in the mouth. Antimicrobial agents with poor enteral absorption used for SDD include COL sulphate, neomycin sulphate, gentamicin, and paromomycin. SDD and SOD can also be combined with a short course of systemic antibiotics (e.g., nitrofurantoin, fluoroquinolones, cotrimoxazole, fosfomycin or erythromycin) [15, 187]. The choice of the topical and systemic antimicrobial agent combinations depends on the resistance patterns, co-occurrence of infection, the targeted colonizing microorganism and institutional preferences.

As anticipated above, routine decolonization of 3GC-R-*Ent* and CRE gut carriers is not recommended by the ESCMID–EUCIC panel [187]. In such analysis, Tacconelli et al. summarized the results of studies performed until August 2017 and considered the effectiveness of the SDD measuring either microbiological or clinical outcome or both. However, we note that the analysis included 19 studies in which SDD was used to decolonize MDR-*Ent* carriers, of which only 2 were randomized controlled trials (RCTs).

In the first RCT (2008-2010), Saidel-Odes et al. administered for 7 days an oral gel with gentamicin and COL sulphate (0.5 g, 4×/day) and an oral solution of gentamicin (80 mg, 4×/day) and COL (1 M units, 4×/day) to 20 patients with CR-*Kp* gut carriage. After 2 weeks, the rate of CR-*Kp* colonization was significantly reduced compared to the placebo arm consisting of 20 patients (61.1% vs. 16.1%, respectively; *P* < 0.0016). A difference between the 2 arms was still maintained at 6 weeks, but with a non-significant difference (58.5% vs. 33.3%, respectively; *P* = NS) [189]. In the RCT of Huttner et al. (2009–2012), 54 patients (27 in each arm) colonized with ESBL-*Ent* received oral COL sulphate (50 mg, 4×/day) plus neomycin sulphate (250 mg, 4×/day) for 10 days. Twenty-eight ± 7 days after the SDD, there was no statistical difference regarding the persistence of ESBL-*Ent* gut colonization between treatment and placebo arms (51.9% vs. 37.0%, respectively; *P* = 0.27) [190].

Although non-randomized, several studies included in the Tacconelli’s analysis deserve to be mentioned. In a retrospective cohort (2012–2015), Machuca et al. analyzed the clinical effect of a 14-day SDD with gentamicin (80 mg, 4×/day) or streptomycin (80 mg, 3×/day) plus neomycin (40 mg, 3×/day) in 44 individuals colonized with COL-R KPC-*Kp*. The authors compared the outcome after 180 days with 33 controls. As a result, gentamicin use resulted in a lower risk of crude mortality [hazard ratios, (HR) = 0.15], lower risk of infection with COL-R KPC-*Kp* (HR = 0.86), and an increased microbiological success (i.e., at least 2 negative rectal swabs after > 48 h after the completion of SDD; HR = 5.67). On the other hand, neomycin plus streptomycin was only associated with a lower risk of mortality (HR = 0.22) [191]. In the retrospective study (2010–2012) of Lübbert et al., 14 patients colonized with KPC-2-*Kp* received a 7-day course of SDD employing oral COL sulphate (1 M units, 4×/day) and gentamicin (80 mg, 4×/day). Decolonization of KPC-2-*Kp* was achieved in 6/14 patients (43%) after a mean of 21 days, but was also observed in 23/76 (30%) of the controls (*P* = 0.102). Of note, SDD treatment resulted in the development of secondary resistance to COL (19% increase in resistance rate) and gentamicin (45% increase) in post-treatment isolates [192]. An increase in aminoglycoside-resistant Gram-negatives was also noted by Oostdijk et al. in 16 Dutch ICUs (2009–2013) implementing the SDD [193].

More recent analyses merit to be cited. In the multicenter RCT of de Lastours et al. (2016–2017), the risk of secondary resistance to COL after its implementation for the SDD was confirmed and the underlying molecular mechanisms of resistance were elucidated [194]. In a cluster-randomized trial (2013–2017) involving 13 European ICUs with 8665 patients, Plantinga et al. showed that SDD (COL plus aminoglycosides) was associated with higher eradication and diminished acquisition of MDR organisms in the rectum compared to the controls (HR = 1.76 and HR = 0.51 for 3GC-R-*Ent*; HR = 3.17 and HR = 0.56 for CR Gram-negatives, respectively) [195]. Döbele et al. assessed the impact of SDD of hematological patients colonized with ESBL-*Ent* on the incidence of BSI after chemotherapy. To do so, a stochastic simulation model was created. The model estimated that decolonization prior to chemotherapy reduces the incidence of ESBL-*Ent* BSI by up to 27%. The greatest benefit was estimated in high prevalence settings, whereas in low-prevalence settings the model estimated no benefit [196].

Overall, in line with the ESCMID–EUCIC panel, we believe that evidence for successful SDD regimens is still limited, mostly because of the lack of well-designed and large/multicenter RCTs with long-term follow-ups. Future studies also need to assess the impact on secondary resistance and disruption patterns in the gut microbiome.

### Fecal microbiota transplantation

The fecal microbiota transplantation (FMT) was initially designed and implemented for the treatment of the recurrent *Clostridioides difficile* infection. It consists in the infusion of liquid stool (via an enteral route, an endoscope, or capsules for ingestion) from a healthy individual into the gut of a patient who suffers from gut dysbiosis. Its mechanism of action is based on the establishment of a new intestinal microbiota community to restore normal gut function [197].

More recently, the FMT has also been shown to be useful for the treatment of other intestinal pathological conditions [198], including the possible eradication of colonization and recurrent infections due to different species of MDROs [199]. In this context, in recent years, the selection and screening of healthy donors together with the collection, preparation and storage of their stools underwent an extensive discussion to reach international standardized procedures. Among them, stool testing must include the search for MDROs (e.g., 3GC-R-*Ent* and CRE) [200]. This is essential to prevent the transmission of MDROs that could lead to adverse infectious events. For instance, DeFilipp et al. described two patients with a BSI due to ESBL-*Ec* after receiving FMT from the same donor. One of the patients died because of severe sepsis [201].

In 2016, Manges et al. reviewed some clinical cases where the FMT was positively implemented to solve gut colonization with ESBL-*Ent* or CPE [202]. For example, a kidney transplant recipient with recurrent ESBL-*Ec* pyelonephritis leading to graft failure underwent FMT to be eligible for re-transplantation. After 1 week, the rectal culture was still positive for ESBL-*Ec*. However, a negative result was obtained at the second week, and subsequent rectal cultures remained negative during the 12-week follow-up period; UTIs were also not observed [203]. After the work of Manges et al., more promising and similar cases were described. For instance, a renal transplant recipient was suffering from recurrent UTIs and BSIs due to an ESBL-*Kp* that was also present in the stool. After several unsatisfactory treatments with meropenem, FMT was implemented and ESBL-*Kp* was not isolated from both urine and fecal samples during the following 8 months [204].

Several small cohort studies have also been performed on this matter. These analyses were mainly uncontrolled and not randomized (summarized in [15, 199, 205]). Moreover, the overall results were not always promising, as speculated in the single case reports mentioned above. Specifically, FMT was successful against colonization due to MDROs in 63% of the case series studies with a control arm. However, a decolonization rate of only 33–46% was observed for MDR-*Ec* and MDR-*Kp* [206]. For example, during 2012-2014, 15 gut carriers of ESBL-*Ent* underwent an FMT showing successful decolonization only in 3 subjects (at 1-, 2- and 4-weeks follow-up). Seven out of the 12 non-responders underwent a second FMT, but only 3 of them resulted decolonized in all time points (after 1-, 2-, and 4 weeks) [207]. In another multicenter prospective study (2015–2017), 8 patients colonized with CRE underwent FMT. As a result, 1 week and 3 months after the FMT, only 3 (37.5%) and 4 (50%) subjects were free of CRE colonization, respectively [208].

In a multicenter RCT (2016-2017), Huttner et al. evaluated whether 5 days of oral SDD with COL and neomycin followed by FMT could eradicate intestinal carriage with ESBL-*Ent* and/or CPE. The primary outcome was the detectable gut carriage of these MDR-*Ent* by culture 35–48 days after randomization. Nine out of 22 (41%) patients who received the treatment resulted negative for ESBL-*Ent*/CPE, while in the control group were 5/17 (29%). As a result, the use of antibiotics plus FMT slightly decreased MDR-*Ent* carriage, but the differences were not statistically significant [209]. In another multicenter RCT (2016–2017), Leo et al. also evaluated whether oral SDD with COL and neomycin followed by FMT could eradicate colonization with ESBL-*Ent* and/or CPE in 16 patients. As a result, 9 (56%) treated carriers and 3 (33.3%) out of 9 controls were considered decolonized 35–48 days after randomization. Metagenomic analyses indicated that antibiotic treatment resulted in a significant change in microbiota composition with reduced species richness and diversity, lower *Firmicutes*/*Bacteroidetes* ratio, decreased proportions of *Proteobacteria* and *Enterobacteriaceae*, and an increase of ARGs abundance. This effect was transient, with a post-FMT microbiota significantly enriched of *Bifidobacterium* species and *Collinsella aerofaciens*, which likely limited the gut colonization by MDR-*Ent*. In contrast, the proportion of *Enterobacteriaceae* in the post-FMT microbiota was lower compared to the baseline (but without statistical significance). Finally, both ESBL and carbapenemase genes were more abundant at baseline than at any later sampling point in 10 out of 16 cases [210].

Further, authors have also attempted to understand the molecular mechanisms responsible for the positive implementation of FMT. In 2019–2020, Lee et al. investigated with 16S rRNA sequencing the dynamic changes of microbiota before and after the use of FMT to decolonize 10 CPE carriers. The rates of the decolonization were 40%, 50%, and 90% within 1, 3, and 5 months, respectively. A significant alteration was observed in the gut microbiota following FMT, but this was different between early decolonization carriers (within 4 weeks) and late decolonization carriers. In fact, before FMT, the early decolonized patients possessed a higher relative abundance of *Bacteroidetes* and showed a microbiota convergence with that of their donors within 4 weeks. Of note, the genera *Hungatella* was only detected in the late decolonization carriers. The authors concluded that molecular characterization of the microbiota of CPE carriers could predict the outcome of FMT and also determine if repeated FMTs are needed [211]. In a recent prospective analysis (2018–2019), Haggai et al. administered oral capsulized FMT for 2 days (15 capsules per day) to 13 CPE carriers. At 1 month, CPE eradication was successful in 9 (69%) patients; 10/13 participants were retested after 6 months and 8/10 of them were negative. Shotgun metagenomic sequencing indicated that bacterial communities showed significant changes in both alpha- and beta-diversities for patients who achieved CPE eradication than those who underwent failure. Notably, in post-FMT samples, beta-diversity analysis identified sample clustering according to treatment outcome. In post-FMT samples, the abundance of *Ent* decreased in responders and increased in non-decolonized subjects. The post-FMT microbiota of responders was compositionally similar to that of donors, whereas that of non-responders was different and rich of ARGs [212]. In another study (2018), Liu et al. analyzed the longitudinal dynamics of the gut virome and bacteriome in 3 recipients who were successfully decolonized from CRE (two carriers of CR-*Kp* and one with both CR-*Kp* and CR-*Ec*) with two FMTs. After FMTs, the gut microbiota changed greatly and resembled that of the donor, especially when the *Ruminococcus* genus was dominant. Furthermore, *Klebsiella* phages expanded with a concordant decrease in *Klebsiella* spp. and increase in *Escherichia* phages in the CR-*Ec* carriers. This may indicate that bacteriophages brought by the FMT may play a key role in MDR-*Ent* decolonization (see next section) [213].

Overall, the currently available information regarding the use of the FMT to decolonize gut carriers of MDR-*Ent* indicates that this approach may have beneficial effects on intestinal carriers. However, as already noted by ESCMID–EUCIC [187] several years ago, no definite suggestions can be made. This is mainly due to the limited number of studies on this matter and the lack of standardized protocols. Moreover, these studies have serious limitations, including the lack of true controls and long-term safety data [206]. Therefore, randomized clinical trials involving large sample sizes and consensus on standardized protocols are warranted. In this context, we note that several RCTs are ongoing (https://clinicaltrials.gov/ct2/home).

### Bacteriophages

Bacteriophages are the most abundant bacterial predators [14]. As evolving and self-replicating biological entities, they benefit from a unique nature compared to traditional antibacterial drugs and are now recognized as a crucial potential alternative in the global fight against antimicrobial resistance (AMR) [214, 215]. They have been used since the 1920s in the former Soviet Union countries and are a valuable prescription-free element of the standard medical practice in this part of the world [216]. In Western countries, the onset and exacerbation of the AMR crisis, combined with recent technological advancements, have provided a boost to the renaissance of phage therapy research [217].

Study reporting on their investigation to treat MDR bacterial infections, either alone or in combination with antibiotics, are now numerous and a great proportion of them show encouraging results [217]. However, scientific articles on their use for decolonization of intestinal carriage are less numerous and even rarer are studies specifically addressing phage-based-decolonization of MDR-*Ent*. A discrepancy partially explained by the divergences in the study of phage-based decolonization in vivo compared with phage-based treatment of infections*.* In fact, during the latter, inflammatory processes caused by the bacterial infection stimulate an immune response, which in turn plays a pivotal role in supporting phage action in clearing the infection [83, 217]. Notably, these host-mediated supportive proinflammatory responses are also involved in facilitating the clearance of phage-resistant mutants, which are often less virulent than their susceptible counterparts. In their absence, as in intestinal colonization, phage-resistant mutants often rapidly emerge after treatment [218].

In this regard, we can highlight the study of Feng et al. [218]. In their murine model, a stable colonization with an ST11 CR-*Kp* was established with a continuous administration of meropenem in drinking water as pre-treatment for 3 days. The targeted strain was then challenged with two lytic phages, alone or in combination, isolated and characterized in the same study. Phage-resistant mutants were characterized by reduced virulence, diminished capsule production, and no change in antimicrobial susceptibility. In this case, phage resistance mechanisms were attributed to capsule polysaccharides and exopolysaccharide coding genes. Moreover, the combination of the two phages (vs. monophage administration) showed a higher and faster reduction in CR-*Kp* count with no development of adverse events. Notably, the two phages were not administered through the same route. One was given orally, while the other—not detectable in the feces—via enema, possibly introducing a methodological bias. Additionally, in a clinical situation where multiple CR-*Kp* strains colonize the intestine, a more complex cocktail may be necessary. On this regard, multiple rounds of phage isolation from bacterial phage-resistant mutants would need to be considered to maximize the targeted lytic activity. These considerations as well as further limitations were extensively discussed by the authors [218]. Noteworthy, we also observed the emergence of phage-resistant mutants using a bioreactor system simulating an intestinal colonization with a ST131 CTX-M-15-*Ec* challenged with a phage cocktail. Interestingly, using the in vitro continuous culture system, we observed an individual-related tendency in the emergence of phage resistance, which might depend on the particular flora of the individual [219].

Researchers from the Institute Pasteur focused on the impact of phage-based decolonization on the intestinal flora [220]. In 2016, they reported encouraging results on the in vitro and in vivo efficacy of three lytic bacteriophages against an antibiotic-resistant uropathogenic *Ec* (AR-UPEC) strain. Bacteriophages, isolated and characterized in the same study, were used as both single therapy and as a cocktail in an experimental murine model. A continuous antibiotic pressure was not required in order to maintain high levels of colonization (the antibiotic was removed from drinking water 3 days before treatment start). Gut carriage levels and the impact of phage treatment vs. antibiotic treatment on the microbiota composition defined the two study outcomes. Seven days after phage treatment start, the AR-UPEC strain showed a distinct decrease in different gut sections, and the same results could be replicated with a 100-fold higher dose in only 4 days. Notably, in this model, the level of intestinal carriage was higher than the ones described in humans colonized with the same strain (i.e., possible weaker efficacy when administered in a clinical setting). The authors also observed that the bacterial count and phage titre decreased at the same rate, providing further evidence for phage’s self-clearing property in the absence of the host. In regard to the effects on microbiota composition, antibiotic treatment disturbed, at a much higher level, its diversity (based on 16S rRNA sequence analyses), confirming the valuable, highly targeted effects of some bacteriophages [220]. Moreover, they observed an increase in the genus *Barnesiella* after phage treatment, which was previously associated with a decrease in vancomycin-resistant *Enterococcus faecium* colonization [221]. This observation opens the discussion—not deepened in this review—on the implementation of phages to restore the healthy microbiota as a microbiome-based decolonization approach as with prebiotics, probiotics or symbiotics (i.e., strengthening colonization resistance) [220, 222–224].

On this regard, Wang et al. recently investigated the therapeutic effect of the combined administration of phage-cocktail and FTM to treat *Salmonella enterica* Typhimurium-induced mouse colitis, compared to phage treatment and to FMT alone [225]. The cocktail was composed of 2 phages lytic for serotypes O4 and O9 isolated from sewage and yak feces from Tibet and belonging to the *Siphoviridae* family. The effect of the combined therapy was evaluated by gavaging fecal matter (or phosphate buffered saline for the control group) 3 h after a single dose of phage cocktail, after which FMT was given at 12, 24, and 36 h after infection. The results clearly showed that the combined therapy phage-FMT was superior to both single treatments. Notably, after 72 h, *Salmonella *Typhimurium was completely eradicated, clinical symptoms of colitis and pathological damages reduced significantly, and the intestinal barrier and short-chain fatty acid levels recovered. Moreover, analysis of the species richness and diversity showed a shift towards a healthy microbial diversity, including the genus *Lactobacillus*. This latter has been reported to play a pivotal role in reducing inflammation during colitis in mice, and the prebiotic effect of its increase may be strongly related to treatment success. Noteworthy, prior to implementing this study design, the authors attempted to treat the same mouse model first exclusively with the two combined phages and then exclusively with FMT. In the first case, they were able to show an initial reduction of bacterial count in the colon, but without completely eradicating the pathogenic strain in the long-term and without completely restoring the complex diversity of the intestinal microbiota. In a follow-up investigation, the authors attempted to use FMT as a single treatment, but again without success. Bacterial counts in the colon remained high, as did inflammatory damage, and no significant recovery of the intestinal microbiota was observed. These initial results, together with the outcome of the combined therapy, suggest that both roles—(i) elimination of the pathogen by the phages and (ii) support in restoring the intestinal barrier and microbiota composition by FMT—are necessary for a successful therapy [225].

The challenge of phage therapy as sole treatment strategy was also highlighted by Javaudin et al. in 2021 [226]. In this French study, the authors explored the efficacy of 4 lytic bacteriophages against an ESBL- and OXA-48-*Ec* in two distinct mouse models of intestinal colonization. Phages were first isolated and characterized, then administered orally and rectally as microencapsulated and non-microencapsulated particles. Colonization models were attained by continuous administration of either amoxicillin or pantoprazole in drinking water, with the latter additionally combined with amoxicillin for the first 8 days. In the first model, phage treatment only transiently reduced the count of the targeted *Ec* strain 9 days after treatment start, while in the second, the targeted strain was not altered at all by the intervention. The use of encapsulated phages did not modify the targeted bacterial count in either case [226].

In the clinic, phage therapy can currently only be used as *extrema ratio* treatment and is therefore mostly administered together with several antibiotics, posing a major problem in the data interpretation. With special regard to decolonization from intestinal carriage with MDR-*Ent*, we can report only on two published case-studies. The first, published in 2019 by Kuipers et al. in The Netherlands and the second in 2020 by Corbellino et al. in Italy [227, 228].

Kuipers et al. reported the successful combined therapy of phages plus meropenem in a 58-year-old renal transplant patient with recurrent UTIs due to an ESBL-*Kp* and an epididymitis [228]. Although susceptible to carbapenems in vitro, the strains could not be eradicated with repeated treatment courses. A urine sample from the patient was sent to the Eliava Institute of Bacteriophages (Tbilisi, Georgia), which in turn sent a personalized phage cocktail for oral ingestion and bladder irrigation. Detailed information on the content of the cocktail, including dose and endotoxin concentration, was not provided by the Eliava Institute. Upon delivery, the lytic activity of the cocktail against the ESBL-*Kp* strain was tested by the authors. Phage treatment was then performed by the patient (i.e., bladder irrigation via catheter). The urethritis symptoms diminished within the first days of treatment, rapidly disappeared, and did not recur. Urine cultures remained negative for ESBL-*Kp* (tested for up to 14 months), and no adverse events were reported [228]. Notably, the patient’s epididymitis was treated in parallel with meropenem for 6 weeks, unfortunately hampering the extrapolation of data on phage effectiveness as a sole treatment strategy.

Corbellino et al. administered a personalized phage treatment to a 57-year-old patient with a high risk of recurrent invasive infections due to a long-standing multi-site colonization (i.e., in the gastrointestinal and urinary tract, as well as in a permanent ureteral stent) with a ST307 KPC-3-*Kp* [227]. Antibiotic cycles with ceftazidime-avibactam (CZA) (still active toward the MDR-*Kp* isolates) showed to be unsuccessful. Five *Kp* isolates from urine, rectal swab and ureteral stent were sent to the Eliava Institute. There, a personalized cocktail of lytic phages was prepared over 9 weeks. The patient collected the preparation in person and received instructions for use. The treatment included a 3-week course of the cocktail by oral and intra-rectal routes. Two weeks after treatment, the ureteral stent was replaced and remained MDR-*Kp* free. The strain was also not detected in the feces, rectal swabs, and urine. Attempts to detect carbapenemase genes from rectal swabs by molecular methods also failed. Notably, following phage therapy, 4 further complicated UTIs and one sepsis occurred. However, in all these 5 distinct episodes, the KPC-3-*Kp* never reappeared. Despite these promising results, the authors remained cautious about judging the cause of MDR-*Kp* eradication. For instance, they pointed out that because of the half-life of CZA combined with reduced creatinine clearance (due to the patient’s solitary kidney), a possible synergy between phages and the antibiotic cannot be excluded. In fact, although phages were given in this case as the only treatment, CZA concentration in urine and blood was not measured upon phage treatment start [227]. Moreover, spontaneous decolonization cannot be excluded (see above).

In conclusion, the clinical application of phages, including personalized phage therapy, still needs to overcome a number of concerns and technical barriers to be considered an effective decolonization strategy [227]. Particularly, high-quality data on safety and efficacy from RCTs are crucial to determine their microbiological, epidemiological, and clinical outcomes [214, 227, 229]. This will include a better assessment of the development of phage resistance, their possible transfer of undesirable genes, their interaction with our immune system and microbiome [214, 217, 230–233]. Lastly, complex pharmaceutical regulatory requirements must be clarified [233]. In particular, obstacles to their production and use in the European Union and the UK must be overcome through new regulations; furthermore, incentives must be created for pharmaceutical companies to increase their interest in this still uncertain area [214]. Notably, personalized phage therapy still falls into the category of new infection control measures, which entails a long and complicated approval process [234]. Only when these challenges will be consistently addressed, phages will be able to contribute in a major way to the global fight against the emergence and selection of new resistant bacteria, including their use as a valuable, well-studied and safe decolonization alternative [219, 227, 233].

### Probiotics

Probiotics are food supplements containing alive bacteria or fungi that are intended to be ingested and reach the intestinal tract (mainly the colon) intact. Most probiotics derive from fermented foods (e.g., yogurt, cheese) that contain a large quantity of *Lactobacillales* able to replace the initial high concentration of *Ent* in these nutrients thanks to the production of lactic acid. Moreover, probiotic organisms can compete with intestinal pathogens by excreting toxins, antimicrobial compounds (e.g., short-chain fatty acids, microcins) and adherence factors, potentiating the immune system and reinforcing mucosa production [235, 236]. Therefore, probiotics could be implemented as food additives to eradicate the pathogenic and/or MDR-*Ent* colonizing the intestinal tract in humans and animals. This hypothesis is supported primarily by numerous studies in animal models (e.g., mice) in which the oral administration of *Bifidobacterium bifidum*, *Lactobacillus rhamnosus*, *Lactobacillus plantarum*, *Lactobacillus fermentum*, or *Bacillus coagulans* had favorable effects on the elimination of pathogenic *Ent*. Furthermore, probiotics have been positively implemented to prevent and mitigate gut colonization with *Ent* (including MDR-*Ent*) in food animal breeding (e.g., broiler) [237].

In humans, the most commonly administered probiotics used to eradicate the gut carriage of MDR-*Ent* contain lyophilized *Saccharomyces boulardii*, *Lactobacillus*, or *Bifidobacterium* species (individually or in various combinations) [236]. For instance, in the study of Ramos-Ramos et al. (2010–2014), 8 long-term carriers of OXA-48-*Ent* received for 3 weeks a daily oral administration of a prebiotic (lactitol; Emportal^®^) plus a probiotic (*B. bifidum* and *Lactobacillus acidophilus*; Infloran^®^). During the study period, all patients showed a relative reduction on the OXA-48-*Ent* intestinal loads. However, at weeks 3, 6, and 9, only 4, 6, and 3 patients had negative OXA-48-*Ent* cultures, respectively [238]. In an RCT (2017–2019), Ljungquist et al. administered twice a day for 2 months a probiotic mixture of 8 different living bacteria (Vivomixx®) to 40 outpatients who were colonized with ESBL-*Ent* for at least 3 months. At the end of the trial, only 5 (12.5%) of the patients had achieved successful eradication of ESBL-*Ent* (i.e., 3 consecutive negative cultures at 3, 6, and 12 months follow-up) [239]. In a LTCF, Zollner-Schwetz et al. evaluated the impact of the multispecies probiotic OMNi-BiOTiC® 10AAD on the intestinal and inguinal skin colonization due to MDR Gram-negatives in 12 patients (including 8 with *Ec* and 3 with *Klebsiella* spp.). At the end of probiotic treatment (week 12), 9/12 patients were still colonized; furthermore, at weeks 20, 24, and 36, patients colonized were 5/12, 5/12, and 8/12, respectively. Analysis of the fecal microbiome at the beginning and at the end of treatment displayed statistically significant growth of the genus *Enterococcus* [240].

Probiotics have also shown their inability to prevent the intestinal colonization with MDR-*Ent*. For instance, in the RCT by Wieërs et al. (2017–2019), 120 elderly patients who received amoxicillin-clavulanate for 10 days were treated for 30 days with placebo, *S. boulardii* CNCM I-745® or a probiotic mixture containing *S*. *boulardii*, *L*. *acidophilus*, *Lacticaseibacillus paracasei*, and *Bifidobacterium lactis* (Bactiol duo®). The prevalence of colonization with ESBL-*Ent* increased at the end of the antibiotic treatment in the placebo, *Saccharomyces* and probiotic mixture arms from 10.3%, 7.7%, and 23.1% to 15.4%, 16.7%, and 27.8%, respectively (*P* = NS). The colonization rates were normalized to the initial values ~ 61 days after the first dose of antibiotics (11.1%, 8.0%, and 19.2%, respectively) indicating no significant differences in the 3 arms [241]. In another RCT (2014–2017), Danish adults traveling to India for 10–28 days received either *L*. *rhamnosus* (Dicoflor®) or no probiotics during their overall journey (both arms, *n* = 30). As a result, preventive treatment with the probiotic had no effect on the occurrence of ESBL-*Ent* colonization, with the incidence being the same in both randomization groups [242].

In conclusion, in contrast to data from animal studies, probiotic supplements appear to only reduce abundance, but not to completely eradicate MDR-*Ent*. We also emphasize that although the use of probiotics is well tolerated, it also carries certain risks [236]. In fact, some patients with underlying diseases (e.g., certain inflammatory bowel diseases), immunocompromised, or with predisposing conditions (e.g., central lines and other permanent indwelling catheters) were reported to develop a *Lactobacillus* spp. BSI and/or endocarditis after receiving a probiotic containing these organisms (e.g., [243–245]).

### Siderophore-microcins

Microcins (Mcc) are low molecular mass (< 10 kDa) antimicrobial peptides (AMPs), usually secreted by *Ent* (mainly *Ec*), that have the capacity to inhibit other bacteria. In some cases, the AMP is post-translationally modified by the linkage of a siderophore moiety derived from enterobactin. These siderophore-Mcc can enter and kill bacteria by mimicking iron–siderophore complexes as a “trojan horse.” So far, four siderophore-Mcc have been described: MccE492, MccH47, MccI47, and MccM. The MccE492 is produced by *Kp*, while MccI47 is found in *Ec*. Both MccH47 and MccM have been reported in phylogroup B2 *Ec*. Overall, Mcc have a role in microbial competitions within the intestinal microflora by exerting potent antibacterial activity against phylogenetically related bacteria [246, 247]. Basically, this is the strategy that pathogenic *Ent* use to overcome the autochthonous gut flora and to colonize the gastro-intestinal tract. For instance, UPEC strains (phylogroup B2) chromosomally produce MccH47 and MccM to emerge and dominate in the gut as a prerequisite to generate subsequent UTIs [246].

On the other hand, production of Mcc may in turn be implemented as a therapeutic option against the pathogenic *Ent.* For example, the oral preparation of strain *Ec* Nissle 1917 is historically used as a probiotic for the treatment of bacterial intestinal diseases. In a mouse model, it was shown that administration of strain *Ec* Nissle 1917 was able to limit the growth of adherent-invasive *Ec* and *Salmonella enterica* in the gut, whereas its mutant (not secreting MccH47 and MccM) was unable to do so [248]. In a recent study, Mortzfeld et al. purified the siderophore MccI47 and showed its potent in vitro activity against MDR-*Ent*, including ESBL-*Ec* and KPC-*Kp* strains. More importantly, they engineered a Nissle 1917 *Ec* strain with a plasmid expressing MccI47. Then, the recombinant *Ec* was administered to mice, which showed the capacity to significantly reduce the amount of KPC-*Kp* colonizing the gut [249]. In another study, the ability of a bacteriophage cocktail and a genetically modified *Ec* strain Nissle 1917 producing Mcc-C7 (probiotic) to reduce gut colonization due to an ST131 *Ec* in a murine model was evaluated. ST131 *Ec* was administered on day 0, while treatment was administered on days 0, 3, and 5. When administered together, the two strategies showed synergistic activity against ST131. Specifically, fecal count was significantly reduced on days 1, 4, and 7; however, on day 10, the count for ST131 was again comparable to the control [250].

Overall, these preliminary studies demonstrate the potential of certain microcins for modulating the gut flora. This is a fundamental step towards the use of engineered probiotics and live biotherapeutic products aimed to selectively remove MDROs from the intestinal tract. Investigations into the optimization, scale-up, and manufacturing of these next-generation therapeutic agents will be needed before entering human trials.

### CRISPR-Cas-like methods (microbiome editing)

CRISPR-Cas-based (clustered regularly interspaced short palindromic repeats) technologies are attractive choices for the development of next-generation antimicrobials [251]. Since its revolutionary conception, CRISPR-Cas-based technologies have found their way to the field of AMR. Specifically, various efforts to fight resistant organisms with CRISPR-Cas technology have been proposed, which take advantage of unique delivery systems [252]. For example, CRIRPS-Cas technology can be advantageous as it can be coupled with specific delivery systems to target specific bacterial species (e.g., delivery of the CRISPR-Cas system to *Ec* using bacteriophages or conjugative plasmids), and to specifically target ARGs once inside a host bacterium (e.g., CRISPR-Cas targeting the region(s) of an ARG) [252].

In vivo targeting of *Ec* and other MDR-*Ent* has been conducted in mouse models with bacteriophage M13 and conjugative plasmids delivery systems [253, 254]. Similarly, both systems have been used as well to target enterohemorrhagic *Ec* in the *Galleria mellonella* insect model [255]. In other insect models, CRISPR-Cas systems have been used to target, for example, the *ompA* gene of *Cedecea neteri* (rare Gram-negative) in *Aedes aegypti* (yellow fever mosquito) [256], and an adhesion gene in *Snodgrassella alvi* important for the gut colonization in bees [257].

A precise methodology to deliver the CRIPR-Cas system and to target specific ARGs (or other sequences; e.g., replicon sequence sites) takes advantage of highly conjugative plasmids (i.e., suicide plasmids). This approach has been used in vitro, for example, by Reuter et al. to target *bla*_OXA-48_ and *tra* genes important for replication in *Ec* and other *Ent* species [258]. In a similar approach, He et al. showed that this system can be used to simultaneously target IncX4/I2/HI2 plasmids and ARGs such as *mcr-1*, *bla*_KPC-2_, and *bla*_NDM-5_ in *Ec* [259]. In contrast, other studies have developed CRISPR-Cas systems to successfully target in *Ec* only ARGs such as ESBL (e.g., *bla*_CTX-M-14_), carbapenemase (*bla*_NDM-5_) and colistin-resistance (*mcr-1*) genes [259–263].

It is clear that the use of CRISPR-Cas systems for the targeted decolonization of MDR-*Ent* is promising. However, before its implementation in humans, further research using in vivo models is needed to address the main CRISPR-Cas problems such as de novo resistance and off-target effects [264].

## The need of in vivo models

As discussed above, numerous alternatives could be implemented in the near future to attempt decolonizing intestinal carriers of MDR-*Ent*. Some of these approaches have already shown promising results in in vitro experiments that could potentially have a significant clinical impact in human medicine (e.g., the CRISPR-Cas approach, [252]). However, these findings remain to be validated in preclinical in vivo models before they can be used in human clinical trials.

The in vivo mouse model has so far represented the gold-standard to study several aspects linked to the intestinal colonization due to *Ent* pathogens (e.g., [218, 225, 226, 249]). Nevertheless, though this approach exhibits several advantages—such as the gastrointestinal similarities to humans—many strong limitations can be found in terms of cost, societal, ethical, and logistical issues, which can all together generate very long and laborious investigation periods [265]. Therefore, numerous alternative models have been suggested. Among the most prominent there are invertebrates (e.g., *Drosophila melanogaster*, *Caenorhabditis elegans*, *G*. *mellonella*) and *Danio rerio* (Zebrafish), which could provide an innovative, suitable, cost-effective, and highly scalable substitute for the mouse [265]. However, only in *G*. *mellonella* and Zebrafish, a gut colonization model with MDR-*Ent* has been tried so far [266]. In addition, such alternative models do not possess a natural intestinal microbiota similar to that of mammals [267, 268].

Overall, new in vivo models are needed to perform screening and large-scale investigations aimed to study new approaches to decolonize the gut carriers of MDR-*Ent.* In this context, we emphasize that numerous funding calls focusing on the advancement of the 3R (Replacement, Reduction and Refinement) research have been recently launched worldwide [269]. In our laboratory, we are studying and developing a new in vivo model of MDR-*Ent* intestinal colonization using *Zophobas morio* larvae (https://data.snf.ch/grants/grant/206400), following a 3R call in Switzerland. Since these larvae possess a human-like microbiota [270, 271], this model could offer numerous advantages over the murine model (e.g., fewer ethical issues, lower costs, faster results, and no need for an animal experimentation facility).

## Conclusions

Nowadays, many people in hospital and in the community may be colonized with MDR-*Ent* at the intestinal level. Identification of such at-risk people is not a particular problem, especially in high-income countries. Indeed, many valid and rapid diagnostic methods have been developed, and both risk factors and predisposing conditions for colonization are now well known. In contrast, effective strategies to eradicate MDR-*Ent* from the gut are not yet available. Both SDD and FMT may have some beneficial effects, but further RCTs considering also their combination are needed. In addition, the alternative and new decolonization approaches have been evaluated only in vitro or, more rarely, in murine models (mostly for bacteriophages).

We believe that future research should focus on the development of novel decolonization strategies that could be used alone or as a complement to others (e.g., in conjunction with FMT). Their potential should first be evaluated with reliable alternatives and large-scale in vivo model studies before being tested in human clinical trials. The development of these new in vivo models will be a key aspect in this field in the near future.

## Data Availability

Not applicable.
